# Overexpressing Centriole-Replication Proteins In Vivo Induces Centriole Overduplication and De Novo Formation

**DOI:** 10.1016/j.cub.2007.04.036

**Published:** 2007-05-15

**Authors:** Nina Peel, Naomi R. Stevens, Renata Basto, Jordan W. Raff

**Affiliations:** 1 The Gurdon Institute, Tennis Court Road, Cambridge CB2 1QN, United Kingdom

**Keywords:** CELLCYCLE, CELLBIO

## Abstract

**Background:**

Centrosomes have important roles in many aspects of cell organization, and aberrations in their number and function are associated with various diseases, including cancer. Centrosomes consist of a pair of centrioles surrounded by a pericentriolar matrix (PCM), and their replication is tightly regulated. Here, we investigate the effects of overexpressing the three proteins known to be required for centriole replication in *Drosophila*—DSas-6, DSas-4, and Sak.

**Results:**

By directly observing centriole replication in living *Drosophila* embryos, we show that the overexpression of GFP-DSas-6 can drive extra rounds of centriole replication within a single cell cycle. Extra centriole-like structures also accumulate in brain cells that overexpress either GFP-DSas-6 or GFP-Sak, but not DSas-4-GFP. No extra centrioles accumulate in spermatocytes that overexpress any of these three proteins. Most remarkably, the overexpression of any one of these three proteins results in the rapid de novo formation of many hundreds of centriole-like structures in unfertilized eggs, which normally do not contain centrioles.

**Conclusions:**

Our data suggest that the levels of centriolar DSas-6 determine the number of daughter centrioles formed during centriole replication. Overexpression of either DSas-6 or Sak can induce the formation of extra centrioles in some tissues but not others, suggesting that centriole replication is regulated differently in different tissues. The finding that the overexpression of DSas-4, DSas-6, or Sak can rapidly induce the de novo formation of centriole-like structures in *Drosophila* eggs suggests that this process results from the stabilization of centriole-precursors that are normally present in the egg.

## Introduction

The centrosome is the main microtubule-organizing center in animal cells, and it consists of a pair of centrioles surrounded by an amorphous pericentriolar matrix (PCM) [1]. An increase in centrosome number is often associated with cancer and may contribute to tumor progression [2, 3]. Although centrosomes are dispensable for cell division in some systems [4, 5], extra centrosomes can lead to multipolar mitotic spindles and thereby to chromosomal instability, which is a characteristic of many cancers. Centrioles are also required for templating the growth of cilia—conserved structures that have diverse and essential roles in development [6]. It is critical, therefore, that each daughter cell inherits a single centriole pair after mitosis.

Duplication of the centrioles is a highly ordered process that is tightly coupled to the cell cycle, and it usually occurs in close apposition to an existing centriole [7, 8]. Genome-wide RNAi and genetic screens in *C. elegans* have identified five proteins required for centriole duplication—SPD-2, ZYG-1, SAS-5, SAS-6, and SAS-4 [9–16]. These proteins localize to centrioles and act in sequence to orchestrate centriole duplication in the worm embryo. SPD-2 helps recruit ZYG-1 to the centrioles and ZYG-1 then recruits SAS-5 and SAS-6 [17, 18]. The loss of SAS-5 or SAS-6 blocks formation of the central tube of the daughter centriole, an early event in the initiation of centriole duplication. SAS-4 is the last protein recruited and it is required for the addition of singlet microtubules to the perimeter of the central tube [18].

Several of the proteins involved in centriole duplication in *C. elegans* have been shown to have similar roles in other species. Sak and Plk4 are *Drosophila* and human protein kinases that are very weakly related to ZYG-1, and they are both required for centriole duplication [19, 20]. SAS-4 has orthologs in humans (CenpJ/CPAP) and *Drosophila* (DSas-4), and DSas-4 is essential for centriole duplication in flies [4]. The human ortholog of SAS-6, HsSAS-6, is a centriolar protein that is essential for centriole duplication [10, 12]. The function of the putative *Drosophila* ortholog of SAS-6 (CG15524) has not been reported.

Importantly, the overexpression of human Plk4 or HsSAS-6 results in the formation of extra centriole-like structures in cultured cells [10, 20], which, in the case of Plk4, have the ultrastructural appearance of centrioles [20]. Thus, the overexpression of at least some of the centriole-duplication proteins is sufficient to drive the formation of extra centrioles, although the mechanism is unknown. It is also unknown whether overexpression of these proteins in vivo can lead to extra centrioles: the production of extra centrioles was not reported when SAS-6 or HsSAS-6 were expressed in *C. elegans* embryos [10, 12].

Although centriole formation usually occurs on a pre-existing centriole that serves as a template, in some circumstances centrioles can form de novo. Such de novo centriole formation is a normal part of the development of some organisms, including the early mouse embryo, parthenogenetic Hymenopteran insects, and the amoeba *Naegleria gruberi* [21–23]. It can also be induced by the ablation of the centrioles in cells that would normally rely on templated centriole production [5, 24, 25]. It seems that many cell types have the ability to form centrioles de novo but that the presence of a single centriole suppresses this pathway [24, 26].

Here, we compare the effects of overexpressing three conserved components of the centriole-duplication machinery in *Drosophila*. First, we confirm that the proposed *Drosophila* homolog of SAS-6 is a centriolar protein that is required for centriole duplication. We then compare the effects of overexpression of DSas-6, DSas-4, and Sak, in vivo. We show directly that the overexpression of DSas-6 drives extra rounds of templated centriole duplication in the *Drosophila* embryo, whereas overexpression in somatic brain cells appears to drive only a modest increase in the number of centrioles. In contrast, overexpression of Sak appears to drive a massive increase in centriole numbers in some, but not all, somatic tissues. DSas-4 does not drive templated centriole overduplication in any tissue. Most remarkably, we show that overexpression of any of the three proteins drives the rapid de novo formation of centriole-like structures in unfertilized *Drosophila* eggs, which normally do not contain any centrioles.

## Results

### DSas-6 Is a Centriolar Protein Required for Centriole Replication in *Drosophila*

**Figure 1:**
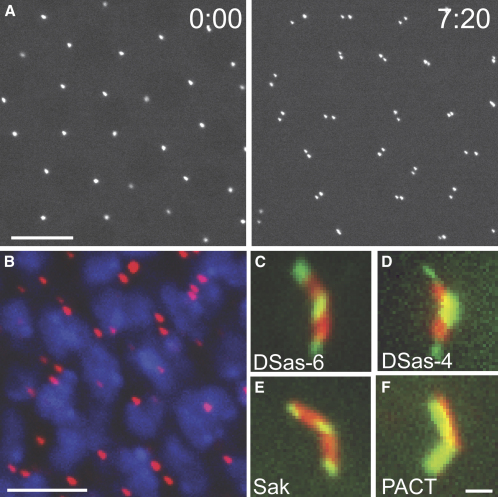
DSas-6 Is a Centriolar Protein (A) GFP-DSas-6 associates with centrioles in living *Drosophila* embryos. Time in min:s is shown in each panel. In interphase (0:00), two centriole pairs are associated with each nucleus, but these pairs can only be resolved as single dots at this stage. These centriole pairs separate from one another in telophase (7:20). (B) The localization of GFP-DSas-6 (pseudocolored red) in whole-mount third instar larval brains. DNA is shown in blue. The centrioles in these cells do not associate with any PCM in interphase [30], so the presence of one or two GFP-DSas-6 dots in every cell indicates that the protein is centriolar, rather than centrosomal. (C–F) The localization of GFP-DSas-6 (C), DSas-4-GFP (D), GFP-Sak (E), and GFP-PACT (F) (green) in spermatocyte centrioles. The centrioles are stained with GTU88^∗^ (red). Note how GTU88^∗^ and GFP-PACT evenly label the entire centriole, whereas the other GFP-fusions are concentrated at the proximal and distal ends. Scale bars represent 10 μm in (A), 5 μm in (B), and 2 μm (C)–(F).

To test whether the putative *Drosophila* ortholog of SAS-6 (CG15524; hereafter DSas-6) is a centriolar component, we raised antibodies against the protein and created transgenic lines expressing DSas-6 as a fusion with green fluorescent protein (GFP) under the control of the ubiquitously expressed ubiquitin (Ubq) promoter [27]. GFP-DSas-6 associated with centrioles at all stages of the cell cycle in embryos, larval brain cells, and spermatocytes (Figures 1A–1C), and DSas-6 antibodies weakly stained centrioles at all stages of the cell cycle in embryos (Figure S1A in the Supplemental Data available online). From these studies, we cannot rule out the possibility that some GFP-DSas-6 or endogenous DSas-6 is also associated with the PCM. In western blots, the anti-DSas-6 antibodies failed to detect endogenous DSas-6, but specifically recognized the GFP-DSas-6 fusion protein (Figure S2A). Thus, like DSas-4 and Sak [4, 19], DSas-6 appears to be a centriolar protein that is normally expressed at very low levels.

**Figure 2:**
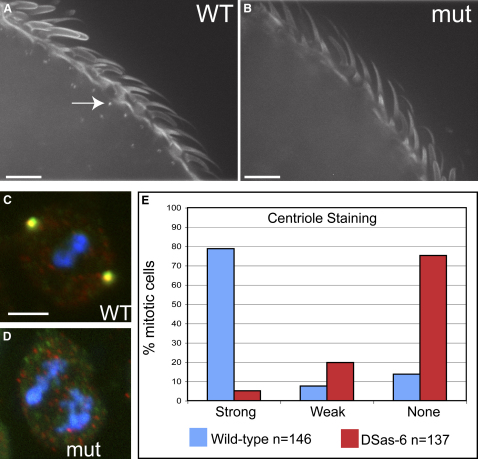
DSas-6 Is Required for Centriole Replication (A and B) The distribution of basal bodies (revealed here with GFP-PACT) in WT (A) and mutant (B) third antennal segments. Basal bodies are present at the base of each sensory bristle in WT antenna (arrow) but are undetectable in mutant antenna. (C and D) The localization of the centriolar marker DSas-4 (red) and the PCM marker Cnn (green) in WT (C) and *DSas-6* mutant (D) mitotic larval brain cells. No centrioles or centrosomes are detectable in the mutant cells. (E) Quantitation of centriole numbers (D-PLP-positive dots) in WT and *DSas-6* mutant mitotic cells (see also Figure S3). More than 70% of mutant cells have no detectable centrioles. Scale bars represent 10 μm.

To test whether DSas-6 is required for centriole replication, we identified a *piggyBac* insertion within the coding region of the *DSas-6* gene (*DSas-6^c02901^*) (Figure S3A) [28]. Approximately 75% of *DSas-6^c02901^* mutants that were transheterozygous with a deficiency that uncovers the *DSas-6* gene died as pharate adults (see Experimental Procedures). The 25% of flies that eclosed were morphologically normal but were uncoordinated, a phenotype often associated with a lack of cilia in the mechanosensory neurons [4, 29, 30]. We confirmed that mutant flies lacked basal bodies and that the majority of mutant third instar larval brain cells lacked centrioles and centrosomes (Figure 2; Figure S3). The uncoordinated phenotype was rescued by the coexpression of the Ubq-GFP-DSas-6 transgene. Thus, like Sak and DSas-4 [4, 19], DSas-6 appears to be essential for centriole replication in flies.

We noticed that GFP-DSas-6 was concentrated at the proximal and distal ends of the large spermatocyte centrioles (Figure 1C), whereas the centriolar markers GTU88^∗^, GFP-PACT, and GFP-Fzr were evenly distributed along the length of the centrioles (Figure 1F; not shown). To test whether this unusual localization was common to proteins required for centriole duplication, we made transgenic lines expressing GFP fusions to DSas-4 (DSas-4-GFP) and Sak (GFP-Sak) under the control of the Ubq promoter. Both proteins had a similar localization to GFP-DSas-6 (Figures 1D and 1E), suggesting that this localization might be important for centriole duplication.

### The Overexpression of DSas-6 Leads to the Overduplication of Centrioles in Embryos

**Figure 3:**
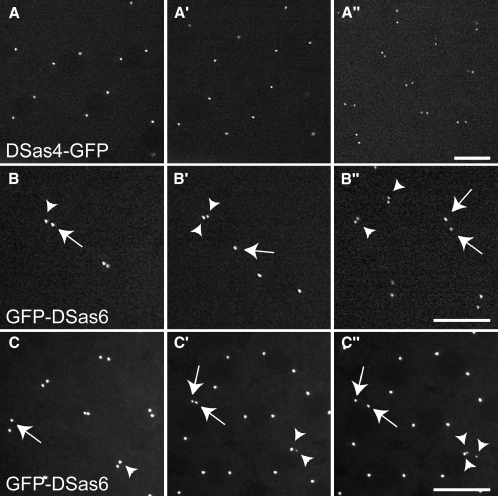
Centriole Overduplication in Embryos Expressing GFP-DSas-6 (A) In embryos expressing DSas-4-GFP, centrioles replicate normally. (B) In the GFP-DSas-6-expressing embryo shown here, the two centrioles marked with an arrow and arrowhead initially separate from one another at telophase (B). During the next interphase, one of the centrioles produces an extra centriole (arrowheads, [B′]). At the next telophase, both of these “extra” centrioles replicate normally and separate in synchrony with the other centrioles in the embryo (arrowheads, [B″]). (C) In the GFP-DSas-6 embryo shown here, two centrioles are highlighted (arrow and arrowhead) at telophase (C). In the next interphase, both of these centrioles produce an extra centriole (arrows and arrowheads, [C′]). The centriole at the bottom right (arrowheads) then replicates one more time to produce three centrioles (C″). See Movies S1 and S2. Scale bar represents 10 μm.

To test whether GFP-Sak, GFP-DSas-6, or DSas-4-GFP could drive the formation of extra centrioles in vivo, we examined early embryos overexpressing these proteins by time-lapse confocal microscopy (TLCM). In these syncytial embryos, each nucleus is associated with a centriole pair at the end of mitosis, and these separate from one another during telophase; the centrioles then immediately start to duplicate as the nuclei enter S-phase (Figure 1A). DSas-4-GFP was concentrated at centrioles, allowing us to directly follow centriole behavior in DSas-4-GFP embryos. We followed >100 individual centriole replication events in 10 embryos (∼1000 replication events in total), and these all proceeded normally (Figure 3A).

GFP-DSas-6 was also strongly concentrated in centrioles, and we followed >100 individual centriole replication events in 15 GFP-DSas-6 embryos (∼2000 replication events in total). Surprisingly, we found that ∼3% of centrioles proceeded through an extra round of template-driven replication within a single nuclear cycle (Figure 3B), and a few of these centrioles proceeded through two extra rounds of replication (Figure 3C; Movies S1 and S2). The extra centrioles appeared to be fully functional because they could proceed through further rounds of replication in synchrony with the other centrioles in the embryo (Figure 3B″). Moreover, the extra centrioles incorporated the centriole markers mRFP-Fzr or mRFP-PACT and, in fixed preparations, they recruited PCM markers (γ-tubulin, D-TACC, and Cnn) and organized microtubules (MTs) (not shown).

Although spermatocyte centrioles were labeled by GFP-Sak (Figure 1E), we were unable to visualize centrioles in embryos with this protein, which suggests that it is present at very low levels. In fixed preparations, we found that the majority of embryos expressing GFP-Sak (60%–80%, depending on the line) arrested early in development with mitotic defects (not shown). To test whether centrioles overreplicated in GFP-Sak embryos, we coexpressed DSas-4-GFP or GFP-Fzr (neither of which, alone, gives mitotic or centriole-replication defects). We observed >1000 individual centriole replication events in 16 embryos, 8 of which had no abnormalities, and 8 of which already had clear mitotic defects. Surprisingly, all centriole replication events occurred normally (Figure S4; data not shown). Thus, the overexpression of GFP-Sak induced mitotic defects in embryos, but we could not directly visualize extra rounds of centriole duplication. The effects of the three proteins on centriole replication in embryos are summarized in Table 1.

### The Overexpression of GFP-Sak and GFP-DSas-6 Leads to the Accumulation of Extra Centriole-like Structures in Larval Brain Cells, but Not in Spermatocytes

**Figure 4:**
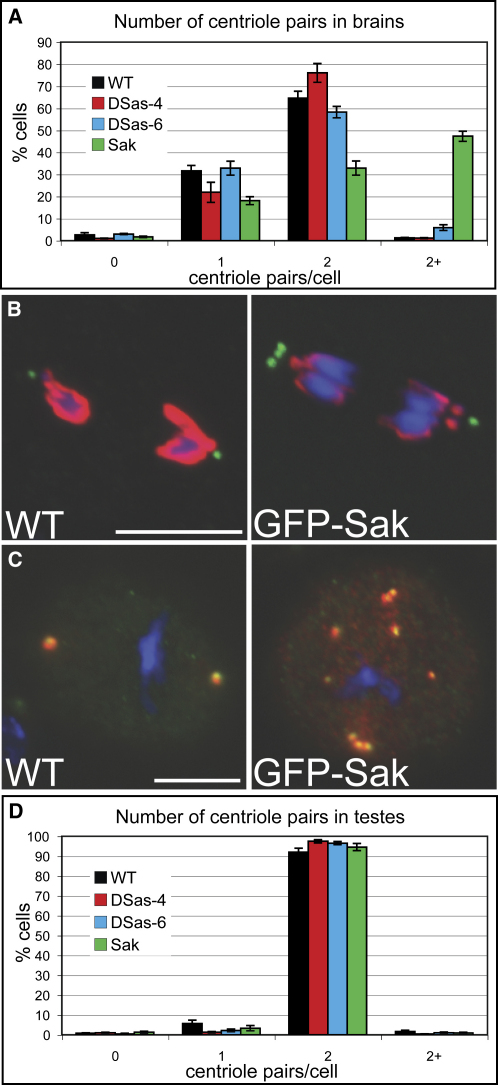
The Overexpression of GFP-DSas-6 and GFP-Sak Drives the Accumulation of Extra Centrioles in Third Instar Larval Brain Cells (A) Quantitation of centriole number (D-PLP-positive dots) in mitotic cells from third instar larval brains expressing different GFP-fusions. For each fusion protein n > 1200 cells (in total) from at least 4 different brain preparations. (B and C) WT or GFP-Sak-expressing mitotic brain cells were stained to reveal centrioles (D-PLP, green) and phospho-Histone H3 (red) (B) or centrioles (D-PLP, green) and γ-tubulin (red) (C). Note how the extra centrioles can cluster at the poles of the spindle (B) and can organize γ-tubulin (C), suggesting that they are at least partially functional. Scale bars represent 10 μm. (D) Quantitation of centriole number (GTU88^∗^-positive centrioles) in spermatocytes expressing different GFP-fusions (as indicated in the panel). For each fusion protein n > 750 cells (in total) from four different testes preparations. Error bars represent the standard error.

To test whether the overexpression of any of the three centriolar proteins could drive centriole overduplication in other tissues, we examined centriole number in mitotic third instar larval brain cells (Table 1). As shown in Figure 4A, the overexpression of GFP-DSas-6 (blue bars) led to a small but significant (p < 0.05) increase in centriole numbers (scored as D-PLP-positive dots), while the overexpression of DSas-4-GFP (red bars) had no effect. The overexpression of GFP-Sak (green bars) caused a dramatic increase in centriole numbers (p < 0.01). Brain cells expressing GFP-DSas-6 or GFP-Sak did not have dramatic mitotic defects, and the accumulation of extra centrioles in these cells was not due to failures in cytokinesis (not shown). Although EM studies will be required to confirm that these extra structures are really centrioles, they were stained by DSas-4 and D-PLP antibodies, and several PCM markers accumulated around them (Figures 4B and 4C; data not shown). Moreover, the overexpression of Sak could not drive the formation of these structures in a *DSas-4* mutant background, demonstrating that DSas-4 is required for their formation (not shown).

We also examined centriole numbers in spermatocytes that expressed GFP-DSas-6, DSas-4-GFP, or GFP-Sak. Surprisingly, we observed no increase in centriole numbers in these cells (Figure 4D; Table 1), even though these proteins appear to be highly overexpressed in testes (Figure S2A).

### High-Level Overexpression of GFP-Sak, GFP-DSas-6, or DSas-4-GFP Drives De Novo Formation of Centriole-like Structures in Unfertilized Eggs

**Figure 5:**
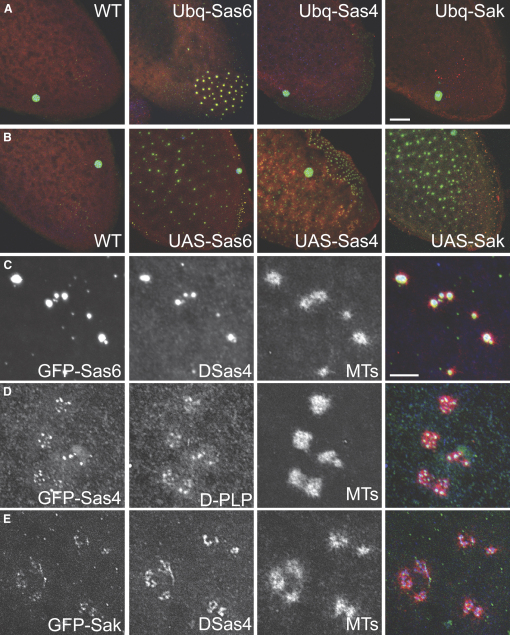
The High-Level Overexpression of Centriole-Replication Proteins Drives the De Novo Formation of Centriole-like Structures in Unfertilized Eggs (A) Unfertilized eggs laid by WT, Ubq-GFP-DSas-6, Ubq-DSas-4-GFP, and Ubq-GFP-Sak females showing the distribution of endogenous DSas-4 (red) and MTs (green). The expression of GFP-DSas-6 can induce the formation of a relatively small number of centriole-like structures. The MT structures in the other eggs are the MTs that surround the polar bodies. (B) Unfertilized eggs laid by WT, UAS-GFP-DSas-6, UAS-DSas-4-GFP, and UAS-Sak-GFP females. All of the eggs are filled with many centriole-like structures. (C and D) Higher-magnification views of the centriole-like structures formed in eggs laid by UAS-GFP-DSas-6 (C), UAS-DSas-4-GFP (D), and UAS-Sak-GFP (E) females. GFP fluorescence is shown in the left panels (green in merged image), endogenous DSas-4 or D-PLP in the middle panels (blue in merged image), and MTs in the right panels (red in merged image). Scale bars represent 25 μm in (A) and (B) and 5 μm in (C)–(E).

To test whether the overexpression of centriole duplication proteins can drive centriole formation de novo, we examined overexpression in unfertilized eggs, which normally do not contain centrioles (see below). We initially screened fixed collections of 0- to 3-hour-old unfertilized eggs for the presence of GFP-containing dots that could organize MT asters (Table 1). We failed to observe any such structures in eggs laid by WT females or females expressing GFP-Sak or DSas-4-GFP (Figure 5A; n > 150 eggs). By contrast, we found that ∼30% (n = 233) of GFP-DSas-6-expressing eggs contained 20–200 large dots of GFP-DSas-6 that organized well-focused arrays of astral MTs (Figure 5A). These structures contained the centriole markers D-PLP and DSas-4, and they organized the PCM markers Cnn and γ-tubulin, demonstrating that they have several characteristics of centrioles (not shown, see below).

In all the experiments described above, GFP-DSas-6, DSas-4-GFP, and GFP-Sak expression were driven by the Ubq promoter, which is expressed at moderate levels in all tissues. Because all of these proteins normally appear to be expressed at very low levels [4, 19, 20], this promoter effectively drives the overexpression of these proteins (Figure S2A). To test whether overexpressing GFP-Sak or DSas-4-GFP at even higher levels could drive the formation of centriole-like structures in unfertilized eggs, we used the UAS/Gal4 promoter system (Figure S2B; see Experimental Procedures). To our surprise, we observed large numbers of centriole-like structures in eggs laid by UAS-GFP-DSas-6, UAS-Sak-GFP, and UAS-DSas-4-GFP females (Figure 5B; Table 2).

Almost all eggs (94/100) laid by UAS-GFP-DSas-6 females contained large numbers (50–1000) of very bright, tightly focused dots of GFP-DSas-6 (Figure 5C). These dots varied in size, and only the larger structures organized MTs and contained the centriole markers D-PLP and DSas-4 and the PCM markers Cnn and γ-tubulin (Figure 5C; data not shown). Almost all eggs laid by UAS-Sak-GFP females (99/100) and the majority of eggs laid by UAS-DSas-4-GFP females (58/100) also contained large numbers (50–1000) of GFP-containing structures, but these were much less bright than the structures observed with GFP-DSas-6, and they usually consisted of clusters of much smaller dots than those seen with GFP-DSas-6 (Figures 5D and 5E). Nevertheless, these clusters all contained the centriolar markers D-PLP and DSas-4 (Figures 5D and 5E) and the PCM markers Cnn and γ-tubulin (not shown).

A qualitative analysis of timed collections of unfertilized eggs suggested that these centriole-like structures formed very early after the eggs were laid (within 5–10 min) and that the numbers of centriole-like structures increased over time only slowly, if at all. To test more directly whether these structures were capable of undergoing rounds of templated replication, we examined the behavior of each GFP-fusion protein in living unfertilized eggs by TLCM. Each GFP-fusion protein exhibited the same characteristic distribution in living eggs as seen in fixed eggs, and, although we followed several eggs of each genotype for more than 30 min (centrioles normally replicate every 8–10 min in syncytial embryos), we never observed any of these structures dividing (data not shown). Together, these findings suggest that the majority of these centriole-like structures form de novo in the eggs and do not undergo templated rounds of replication.

### The Overexpression of GFP-DSas-6, DSas-4-GFP, or Sak-GFP Does Not Prevent the Loss of Centrioles during Oogenesis

We reasoned that the centriole-like structures in these eggs might not have formed de novo, but, instead, might simply be centrioles that had overreplicated and then not been lost during oogenesis. In *Drosophila*, oogenesis begins with 4 rounds of mitotic division that produce a 16-cell cyst; one cell becomes the oocyte, while the others become nurse cells. The majority of nurse-cell centrioles migrate into the oocyte through cytoplasmic bridges in region 2b of the germarium, but a small number remain in the nurse cells [31]. During this migration phase, centriole numbers in the cyst start to decline, and, by the onset of meiosis I during stage 13, all of the centrioles are believed to be lost (reviewed in [31]).

**Figure 6:**
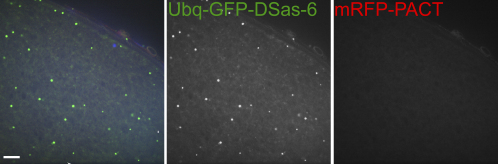
Centrioles Appear to be Inactivated Normally in Oocytes Expressing Centriole-Replication Proteins In stage 14 oocytes, some GFP-aggregates can still be detected in oocytes expressing any of these GFP-fusions (Ubq-DSas-6 is shown here), but none of these colocalize with mRFP-PACT, suggesting that they are not centrioles. Scale bar represents 10 μm.

To examine whether centrioles overreplicated during oogenesis, we counted centriole numbers in the nurse cells (because the oocyte centrioles clustered together and were difficult to count). In wild-type ovaries, the number of centrioles (scored as D-PLP-positive dots) in the nurse cells declined from an average of ∼3 per cyst in region 3 of the germarium to ∼1 per cyst at stage 6/7 (Figures S5A and S5C). In contrast, in UAS-Sak-GFP-expressing ovaries, we observed a dramatic increase in centriole numbers over these stages (Figures S5B and S5D). No such increase was apparent in any of the other Ubq- or UAS-overexpressing lines (Table 2), although GFP aggregates that failed to stain with centriole markers could be observed in all lines (Figures S5E–S5H; data not shown). By stage 14, however, we were unable to detect any D-PLP- or mRFP-PACT-positive dots in ovaries from any of the Ubq or UAS lines, although some GFP aggregates were still detectable in all of the lines (Figure 6; data not shown). Thus, the centrioles appear to be lost normally during oogenesis even when centriole replication proteins are overexpressed.

## Discussion

In this study, we show that DSas-6, like DSas-4 and Sak, is required for centriole duplication in *Drosophila*. Studying the effects of overexpressing each of the three proteins, we show the following: first, the overexpression of DSas-6 in vivo can drive extra rounds of templated centriole replication within a single cell cycle. Second, the overexpression of these proteins induces the formation of extra centriole-like structures to varying extents in different tissues. Third, the overexpression of any of these proteins at high levels can drive the de novo formation of centriole-like structures in unfertilized eggs. We discuss the implications of each of these findings in turn.

It has previously been shown that the overexpression of Plk4/Sak in human cells leads to an accumulation of extra centrioles [10] and HsSAS-6 appears to have a similar effect [10, 20]. Because these experiments were performed with fixed cultured cells, it was unclear how the extra centrioles formed and whether these proteins could drive centriole accumulation in vivo. In our experiments, we have directly visualized extra rounds of templated centriole replication driven by the overexpression of DSas-6 in vivo. Moreover, these extra centrioles appear to be fully functional because they organize PCM and MTs and, most importantly, they can undergo further rounds of replication in synchrony with the other centrioles in the embryo.

Recent studies in *C. elegans* have revealed that centriole replication requires the ordered activity of SPD-2, ZYG-1, SAS-5, and SAS-6, and finally SAS-4 [17, 18]. Our findings demonstrate that DSas-6 levels are critical in determining the number of centrioles formed during centriole replication in *Drosophila* embryos. How might DSas-6 regulate centriole number during replication? One possibility is that, when overexpressed, DSas-6 is recruited normally to the mother centriole but is then inappropriately recruited to the newly formed daughter centriole, thereby inducing the formation of a “granddaughter” centriole. Another possibility is that excessive recruitment of DSas-6 to the mother centriole expands the area where centrioles can form, thereby resulting in the generation of multiple daughter centrioles. Neither mechanism is mutually exclusive, and both of these configurations of centrioles have been observed in *Drosophila* somatic cells in which the inactivation of Cdk1 led to centriole overduplication [32].

We did not directly observe extra rounds of templated centriole replication in Ubq-GFP-Sak embryos, but we suspect that this is because the protein was expressed at very low levels in embryos. In larval brain cells and ovarian nurse cells, Sak was the most potent of the three replication proteins at inducing the formation of extra centriole-like structures. The formation of these extra structures required DSas-4, and the structures contained several centriole markers and could organize PCM markers and MTs. Nevertheless, EM studies will be required to confirm that these structures are true centrioles.

A priori, it is perhaps surprising that two different proteins can drive centriole overduplication, because only one protein would be expected to be rate limiting in any given system. As described above, our data suggest that it is the amount of DSas-6 at the centriole that determines the number of daughter centrioles formed during each round of replication (the “litter” size; [8]), and we suspect that overexpressed Sak can recruit extra DSas-6 to the centrioles even when DSas-6 is not overexpressed. The configuration of the extra centrioles in human cells overexpressing Plk4/Sak is consistent with this proposal, and the formation of these extra centrioles requires HsSAS-6 [20]. Our observation that DSas-4 overexpression does not induce templated-centriole overduplication in any of the cell types we examined is consistent with this hypothesis, because SAS-4 is recruited to centrioles only after ZYG-1 and SAS-6 in *C. elegans* [17]. Overexpressed DSas-4 is presumably unable to recruit extra DSas-6 to the centrioles.

Our results demonstrate that the overexpression of centriole duplication proteins can have different effects in different tissues. The overexpression of GFP-Sak or GFP-DSas-6 leads to an accumulation of extra centrioles in larval brain cells but not in larval spermatocytes. It seems unlikely that these differences result only from differing expression levels in the different tissues, because the Ubq promoter appears to drive higher levels of GFP-DSas-6 and DSas-4-GFP in the testes than in the brain (Figure S2A). We speculate, therefore, that additional mechanisms may regulate the activities of these proteins, and these mechanisms may differ between tissues.

Perhaps the most surprising of our observations is that the expression of any of the three fusion proteins at high levels can trigger the de novo formation of many hundreds of centriole-like structures in unfertilized eggs. EM studies will be required to see whether these structures are normal centrioles, but they all incorporate endogenous centriole markers and organize PCM and astral MTs. Nevertheless, there are clear morphological differences between the structures formed by the overexpression of GFP-DSas-6 and those formed by the overexpression of DSas-4-GFP and GFP-Sak. Interestingly, it has previously been shown that the expression of a dominant mutant form of dynein heavy chain, *Laborc^D^*, can lead to the rapid de novo formation of centriole-like structures in a manner very similar to that reported here [33]. An EM analysis revealed that these structures were “rudimentary centrioles” that consisted of hollow tubes that lacked any associated MTs. The de novo formation of centrioles in cultured cells also leads to the formation of centriole-like structures that, initially, do not have the normal appearance of centrioles at the EM level [25].

Whereas the de novo formation of centrioles in cultured cells is a slow process that occurs over several hours [24, 25], the centriole-like structures that we observe in unfertilized eggs appear very rapidly upon egg deposition. Even in 30 min collections of both UAS-GFP-Sak and UAS-GFP-DSas-6 unfertilized eggs, we found that >95% of the eggs had at least ∼50 of these structures and most had several hundred structures that had already recruited PCM components and were nucleating MTs. Because the expression of these replication proteins does not lead to the abnormal persistence of centrioles during oogenesis, we conclude that the centriolar components in these unfertilized eggs must be organized in such a way that they can be very rapidly assembled into centriole-like structures when the egg is deposited.

This is further supported by our observation that even DSas-4-GFP can induce the formation of centriole-like structures in unfertilized eggs. SAS-4 functions at a late step in centriole duplication [18], so it is unlikely that it could induce the de novo formation of centriole-like structures unless the centriolar components were already partially assembled. We speculate that centriolar components normally have a tendency to transiently self-assemble into “centriole precursors” in these eggs. The overexpression of any of the replication proteins can stabilize these precursors, allowing them to mature into centriole-like structures upon egg deposition.

These observations are consistent with the hypothesis that normal templated centriole replication may depend upon the presence of centriole-precursors in the cytoplasm [24]. In this model, cells normally contain centriole precursors, but during replication only one of these becomes stabilized when it contacts the mother centriole, thereby allowing it to mature into a daughter centriole. In unfertilized *Drosophila* eggs, the overexpression of replication proteins may stabilize these centriole precursors throughout the egg, thereby circumventing the normal requirement that the centriole precursors contact the mother centriole to become stabilized.

## Experimental Procedures

### Generation of GFP- and mRFP-Fusions and Transgenic Lines

*P* element-mediated transformation vectors containing GFP-fusions to Sak, DSas-4, and DSas-6 were generated by initially amplifying the complete coding region of each protein (either from full-length cDNAs, or, in the case of DSas-4, from genomic DNA) with *att* sites at either end for Gateway cloning (Invitrogen). These fragments were inserted into the Gateway pDONR Zeo vector. This was then recombined with either Ubq (R.B., unpublished data) or UASp (T. Murphy, personal communication) plasmids so that each coding sequence was placed, in-frame, with GFP at either their N or C terminus (full cloning details are available upon request). The following transgenic lines were generated by standard *P* element-mediated transformation: Ubq-GFP-DSas-6, Ubq-DSas-4-GFP, Ubq-GFP-Sak; UASp-GFP-DSas-6, UASp-DSas-4-GFP, UASp-Sak-GFP. The Ubq promoter drives moderate levels of expression in all tissues [27], whereas the UASp transgenic lines were crossed to the V32a line, which expresses a Gal4/VP16 fusion protein driven from a maternal tubulin promoter; this drives very high-level overexpression in the female germline [30].

We also used two previously described transgenic lines that expressed GFP-centriolar markers: GFP-Fzr [4, 34] and GFP-PACT [30]. In several experiments we wanted to follow the behavior of two centriolar proteins at the same time, so we generated transgenic lines that expressed mRFP-PACT and mRFP-Fzr (E.P. Lucas, personal communication). These were crossed with GFP-fusion-expressing flies to generate flies expressing both fusion proteins.

### Generation of DSas-6 Antibodies

The following regions of the DSas-6 coding region were amplified by PCR and subcloned, in-frame, into the pMal expression vector (NEB, USA): NT (1–210 aa); Mid (104–317 aa); CT (224–414 aa). MBP-fusions of each region were purified as described previously [35] and antisera were raised against each protein in two rabbits by Eurogentec (Seraing, Belgium). Both the NT and CT antibodies recognized centrioles in embryos, whereas the Mid antibodies worked best in western blotting experiments.

### The *DSas-6^c02901^* Mutation

We obtained the *DSas-6^c02901^*mutation [28] from the Exelexis *piggyBac* collection at the Bloomington Stock Centre. The majority (∼99%) of flies homozygous for this mutation died as pharate adults (where the adults appear morphologically normal, but do not eclose from their pupal cases). This decreased to ∼90% when the mutation was made transheterozygous with the Df(3L)Exel6213 (which uncovers the *DSas-6* gene), suggesting that there were other mutations on the mutant chromosome. We attempted to clean up the chromosome by using recombination with a *ru,st,e,ca* chromosome, but our healthiest stock was still 90% lethal when homozygous and ∼75% lethal when transheterozygous with the deficiency. Even when rescued with the Ubq-GFP-DSas-6 transgene, approximately 60% of flies still died as pharate adults, although the surviving flies were no longer uncoordinated. Because flies that completely lack centrioles and centrosomes are viable, but uncoordinated [4], we suspect that this late lethality is not caused by the *DSas-6^c02901^* mutation. We cannot rule out the possibility, however, that DSas-6 has some function that is unrelated to its role in centriole replication, or that the *DSas-6^c02901^* allele produces a truncated form of DSas-6 that causes this late lethality when homozygous.

### Live Analysis of Centriole Replication in Embryos

Embryos expressing the various GFP-fusion proteins were aligned and observed on a Perkin Elmer ERS Spinning Disc confocal system as described previously [4]. Movies and Figures were also processed and assembled as described previously [30].

### Fixed Analysis of Oocytes, Embryos, Larval Brains, Larval Spermatocytes, and Antennal Segments

Third instar larval brains, testes [4], embryos, and antennal segments from late pupae [30] were fixed, stained, and analyzed as described previously. Oocytes were dissected in PBT and fixed in 4% PFA in PBT for 20 min, stained, and analyzed as described in [30].

### Antibodies

The following antibodies were used in this study: rabbit anti-DSas-6 (described above); rabbit anti-D-PLP [30]; rabbit anti-DSas-4 [4]; DM1a mouse monoclonal anti-α-tubulin (Sigma); rabbit anti-phospho-histone H3 (Abcam); rabbit anti-D-TACC [35]; rabbit anti-Centrosomin (R.B., unpublished data); GTU88 mouse monoclonal anti-γ-tubulin (Sigma); and GTU88^∗^, a batch of the GTU88 monoclonal antibody (Sigma) that crossreacts with centrioles in *Drosophila* [30]. Affinity-purified antibodies were used at 1–2 μg/ml in immunofluorescence and immunoblotting experiments, and all sera or commercial antibodies were used at 1/500 dilutions. Appropriate Alexa 488, Cy3, and Cy5 secondary antibodies were obtained from Molecular Probes or Jackson Laboratories.

## Figures and Tables

**Table 1:** Ubiquitous Moderate Overexpression of Centriole Replication Proteins

	Syncytial Embryos (Templated Formation)	Brain Somatic Cells	Oogenesis Female Germline	Spermatogenesis Male Germline	Unfertilized Eggs (De Novo Formation)
Ubq-GFPSas-6	√	√	×	×	√
Ubq-GFP-Sak	×	√√	×	×	×
Ubq-Sas4-GFP	×	×	×	×	×

√ and √√, Extra centrioles are formed. ×, Extra centrioles are not detected.

**Table 2:** High-Level Overexpression of Centriole Replication Proteins in the Female Germline

	Oogenesis Female Germline	Unfertilized Eggs (De Novo Formation)
UAS-GFP-Sas-6	×	√√
UAS- Sak-GFP	√	√√
UAS-Sas4-GFP	×	√

√ and √√, Extra centriole-like structures are formed. ×, Extra centrioles are not detected.

## Supplemental Data

Document S1. Five Figures

Movie S1. A Field from a GFP-DSas-6-Expressing Embryo The movie starts as the centrioles are separating in telophase, and the arrows highlight two centrioles that will undergo an extra round of replication during interphase. The centriole at the top left undergoes one extra round of replication to produce two centrioles of approximately the same size, whereas the one at the bottom divides twice in quick succession to produce one large centriole and two smaller centrioles.

Movie S2. Continuation of Movie S1 The position of the centrioles that overreplicated during interphase are highlighted with arrows and arrowheads. The top two centrioles (arrows) both duplicate at the end of mitosis, although their separation is slightly delayed relative to the other centrioles around them. The bottom three centrioles (arrowheads) are uneven in size; the two smaller centrioles do not replicate at the end of this round of mitosis (although they went on to replicate at the end of the next round of mitosis). The larger centriole replicates in synchrony with the surrounding centrioles.
