# Supplementary material for: Overexpressing Centriole-Replication Proteins In Vivo Induces Centriole Overduplication and De Novo Formation
Source: Curr Biol. 2007 May 15;17(10):834–43. doi: 10.1016/j.cub.2007.04.036 (PMC1885955; doi:10.1016/j.cub.2007.04.036)
Supplement: Document S1. Five Figures [file mmc1.pdf]

Supplemental Data

Overexpressing Centriole Replication Proteins In Vivo Induces Centriole Overduplication and De Novo Formation

Nina Peel, Naomi R. Stevens, Renata Basto, and Jordan W. Raff

Supplemental References

S1. Bettencourt-Dias, M., Rodrigues-Martins, A., Carpenter, L., Riparbelli, M., Lehmann, L., Gatt, M.K., Carmo, N., Balloux, F., Callaini, G., and Glover, D.M. (2005). SAK/PLK4 is required for centriole duplication and flagella development. Curr. Biol. *15*, 2199–2207.

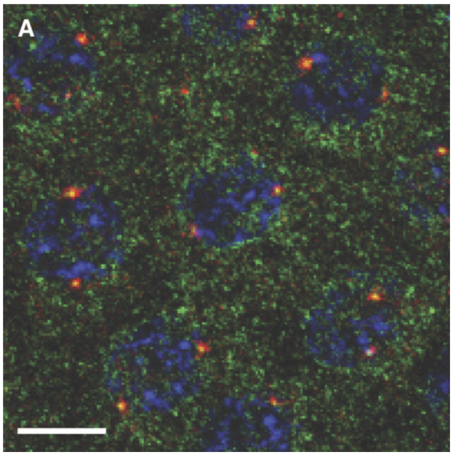

Figure S1. The Endogenous DSas-6 Is Concentrated at Centrioles

WT embryos were stained with DSas-6 (green) and γ-tubulin antibodies (red); DNA is shown in blue. The signal with the DSas-6 antibodies is very weak, but a small dot can be seen within each centrosome. Scale bar represents 10 μm.

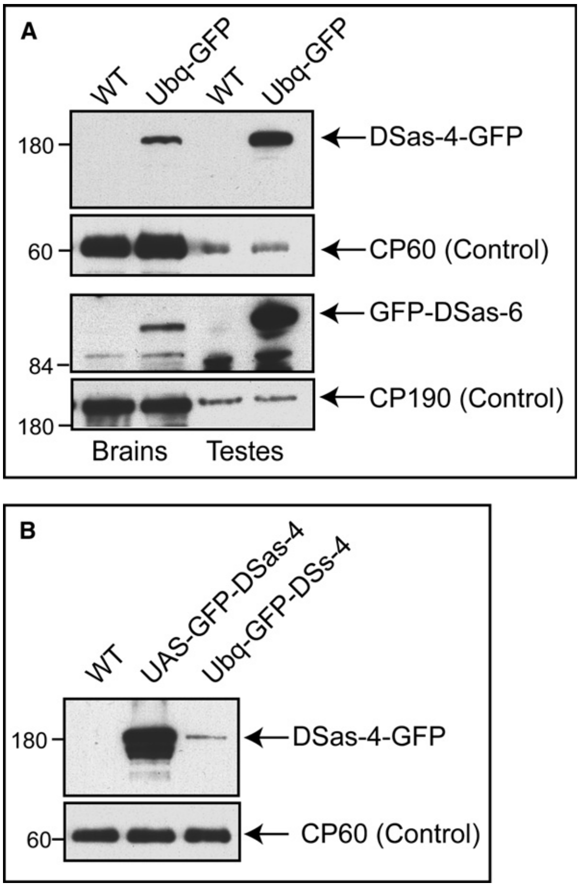

Figure S2. GFP-DSas-6 and DSas-4-GFP Are Overexpressed in Brains and Testes, and the UAS/Gal4 System Leads to a Massive Overexpression of Proteins in Embryos

(A) A western blot of third instar larval brains or larval testes from WT or either Ubq-DSas-4-GFP- or Ubq-GFP-DSas-6-expressing lines. 10 brains and 10 pairs of testes were loaded per lane, and blots were probed with DSas-4 or DSas-6 antibodies, as indicated. Anti-CP60 or anti-CP190 antibodies were used as a loading control. The DSas-4 antibodies recognize DSas-4-GFP, but no band of the correct size for the endogenous DSas-4 is detectable. The DSas-6 antibodies recognize GFP-DSas-6 as well as several background bands that are also present in the WT tissues; none of these appear to be the endogenous DSas-6 because none of them are diminished in intensity in S2 cells that are depleted of DSas-6. Thus, these antibodies can detect the overexpressed GFP-DSas-6 and DSas-4-GFP in brains and testes, but the endogenous proteins are not present at detectable levels.

(B) A western blot of 40 early embryos laid by WT, UAS-DSas-4-GFP, or Ubq-DSas-4-GFP females. This figure illustrates that the UAS/Gal4 system leads to a massive increase in the overexpression of DSas-4-GFP when compared to the Ubq promoter. Again, the endogenous DSas-4 protein is not detectable.

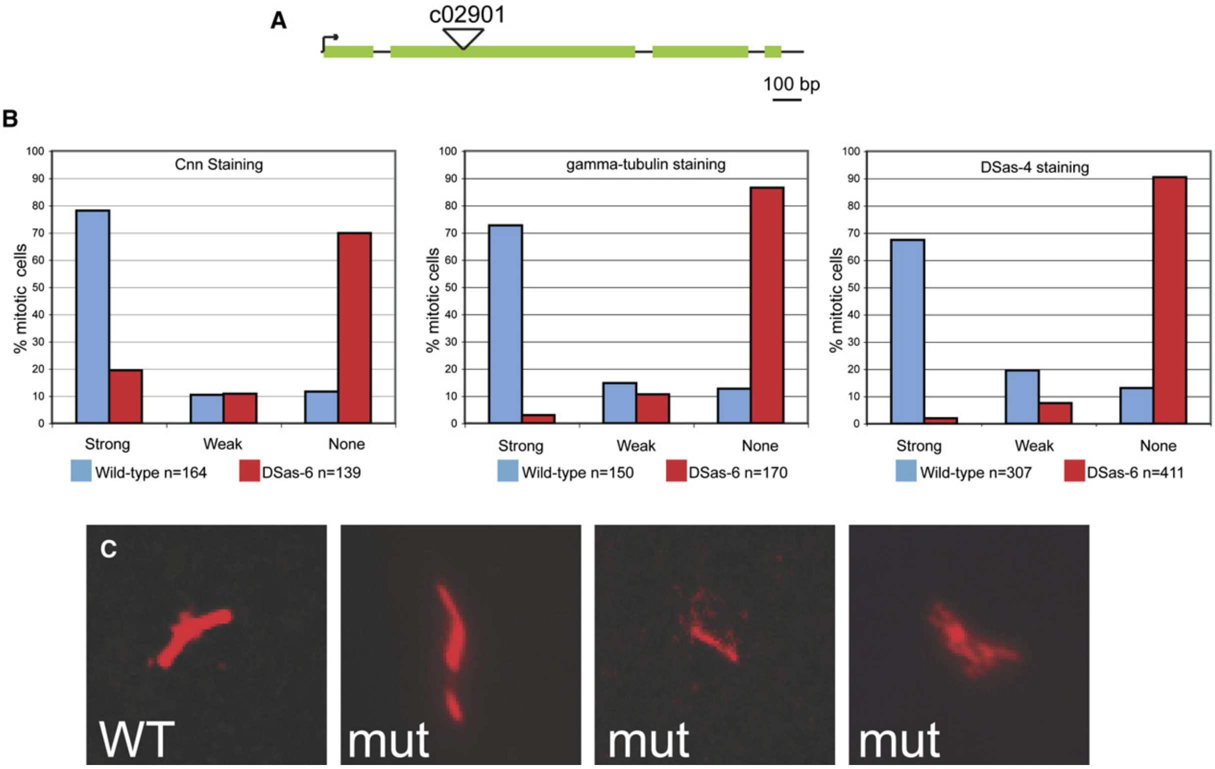

Figure S3. Characterization of the *DSas-6*^{c02901} Mutation

(A) A schematic representation of the *DSas-6* gene (green bars represent exons, black lines introns) showing the position of the c02901 *piggyBac* insertion.

(B) Quantitation of centrosome (as revealed by Cnn or γ-tubulin staining) or centriole (as revealed by anti-DSas-4 staining) numbers in WT and *DSas-6* mutant third instar mitotic larval brain cells.

(C) The centrioles in WT spermatocytes (revealed by GTU88* staining) usually displayed the typical "V" shape arrangement. In *DSas-6* mutant spermatocytes, many centrioles appeared to be fragmented or partially frayed. This is in contrast to the situation in *Sak* mutant spermatocytes where cells either have perfectly formed centrioles or completely lack centrioles [S1]. This suggests that the centrioles gradually lose their normal structure as cells become depleted of DSas-6.

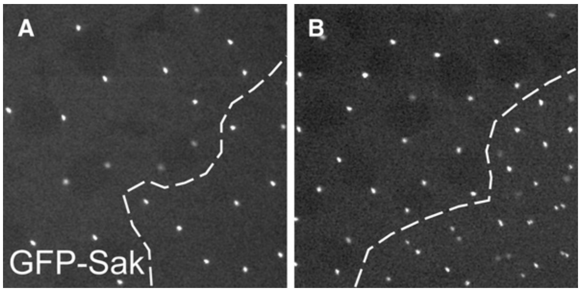

Figure S4. The Expression of GFP-Sak Leads to Mitotic Defects in Embryos, but Extra Rounds of Centriole Replication Are Not Observed

(A) In the GFP-Sak embryo shown here, the nuclei at the top left of the panel are normal, and each is associated with two centriole pairs (revealed here with DSas-4-GFP). At the bottom right of the panel, some defective nuclei have just fallen into the middle of the embryo, leaving the centrioles behind at the cortex. The dotted white line delineates the region between the normal nuclei and the free centrioles.

(B) This embryo proceeded through mitosis, and all of the centrioles replicated synchronously. The centrioles in the top left of the panel remain associated with nuclei, while the centrioles at the bottom right of the panel are not associated with nuclei and so do not separate properly and become bunched together.

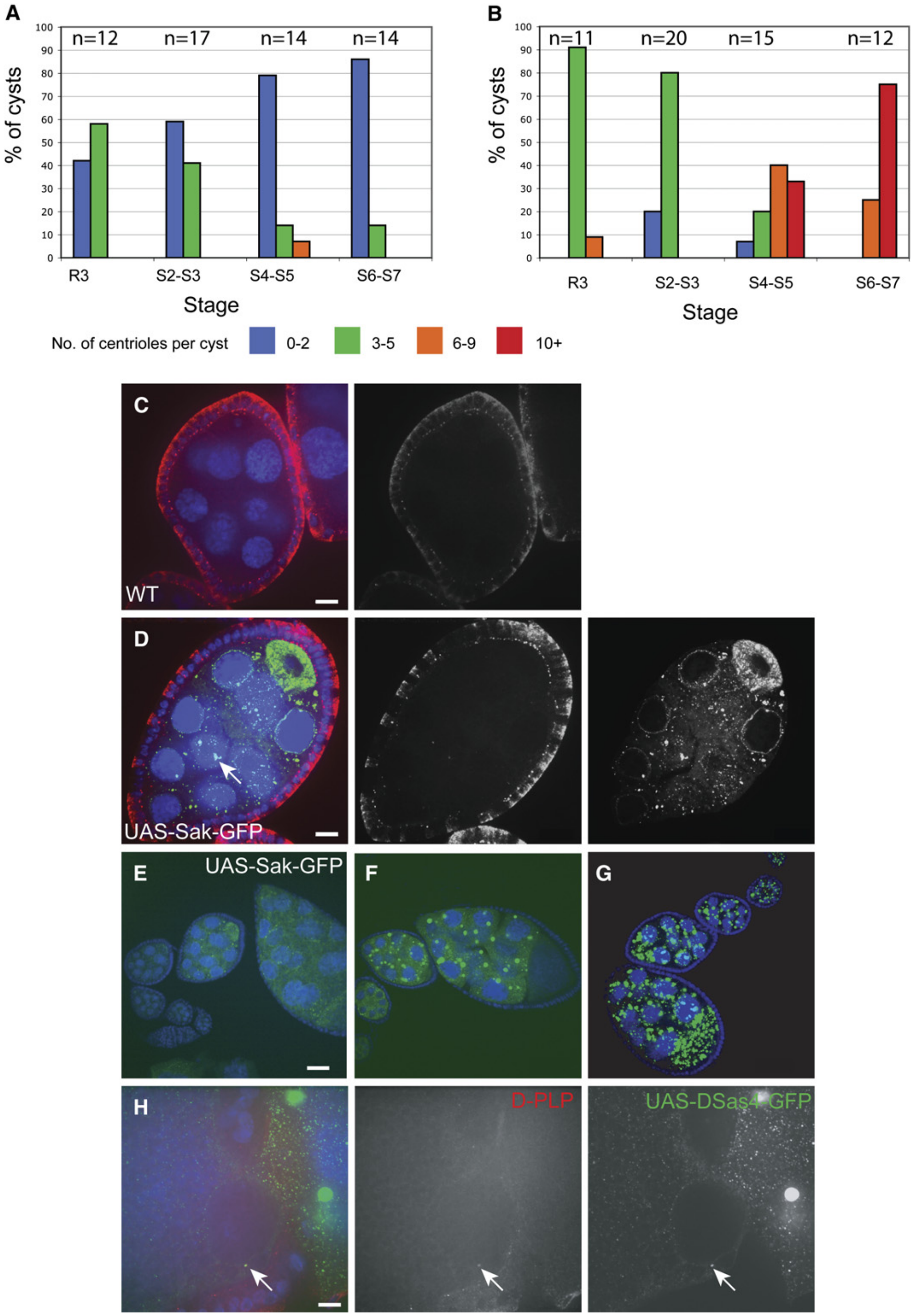

Figure S5. An Analysis of Centriole Behavior in Ovaries Overexpressing Centriole Replication Proteins

(A and B) Quantitation of centriole number (D-PLP-positive dots) in the nurse cells of WT (left) or UAS-Sak-GFP (right) cysts.

(C and D) Immunofluorescence images of a WT (C) and UAS-Sak-GFP (D) cyst. The Sak-GFP (green) forms lots of dot-like structures in the nurse cells and oocyte. Several of these dots are costained with the centriole marker D-PLP (arrow), indicating that they are real centrioles. Note that the centrioles in the surrounding follicle cells are stained by D-PLP antibodies, but Sak-GFP is not expressed in these cells.

(E–G) Ovaries dissected from females expressing UAS-Sak-GFP, UAS-DSas-4-GFP, or UAS-GFP-DSas-6 (green) were stained to reveal the DNA (blue). All of these fusion proteins form prominent aggregates in the cytoplasm of the cysts.

(H) Staining of ovaries with D-PLP antibodies revealed that most of these aggregates were not centrioles. In the egg chamber shown here (expressing UAS-DSas-4-GFP), a large aggregate is present in the nurse cell but it does not contain any D-PLP. Lots of smaller aggregates can be seen in the nurse cell and oocyte, but only a single dot in the oocyte costains with D-PLP (arrow); this dot is associated with the oocyte nucleus, as expected of the real centriole.

Scale bars represent 10 μm in (C), (D), and (H) and 30 μm (E)–(G).
